# A functional MRI pre-processing and quality control protocol based on statistical parametric mapping (SPM) and MATLAB

**DOI:** 10.3389/fnimg.2022.1070151

**Published:** 2023-01-10

**Authors:** Xin Di, Bharat B. Biswal

**Affiliations:** Department of Biomedical Engineering, New Jersey Institute of Technology, Newark, NJ, United States

**Keywords:** functional MRI, head motion, pre-processing, quality control, resting-state, skull stripping

## Abstract

Functional MRI (fMRI) has become a popular technique to study brain functions and their alterations in psychiatric and neurological conditions. The sample sizes for fMRI studies have been increasing steadily, and growing studies are sourced from open-access brain imaging repositories. Quality control becomes critical to ensure successful data processing and valid statistical results. Here, we outline a simple protocol for fMRI data pre-processing and quality control based on statistical parametric mapping (SPM) and MATLAB. The focus of this protocol is not only to identify and remove data with artifacts and anomalies, but also to ensure the processing has been performed properly. We apply this protocol to the data from fMRI Open quality control (QC) Project, and illustrate how each quality control step can help to identify potential issues. We also show that simple steps such as skull stripping can improve coregistration between the functional and anatomical images.

## 1. Background

Functional MRI (fMRI), especially blood-oxygen-level dependent (BOLD) fMRI (Ogawa et al., 1992), has become a popular technique to study brain functions underlying cognitive and affective processes, and to investigate brain alterations in psychiatric and neurological disorders. The sample sizes of fMRI studies have been steadily increasing over the years (Poldrack et al., 2017; Yeung et al., 2020), and many researchers have taken advantages of large open-access datasets, such as 1,000 Functional Connectomes Project (Biswal et al., 2010), autism brain imaging data exchange (ABIDE) (Di Martino et al., 2014), Alzheimer's Disease Neuroimaging Initiative (ADNI) (Jack et al., 2015), and OpenNeuro (Markiewicz et al., 2021). The wide availability and the heterogeneity in acquisition protocols and data quality make it challenging for data processing and statistical analysis. Quality control on the data processing has become a critical component in research but has not been fully charted.

The quality assurance for an fMRI study span from data acquisition to data processing and statistical analysis (See Lu et al., 2019 for an overview). Here we assume that the data have already been collected or obtained from an online repository. Then the quality assurance starts with checking the quality of the images, and mainly involves the data processing steps. There are automated quality control measures for specific steps, e.g., assessing the quality of MRI images (Esteban et al., 2017) and brain registration (Benhajali et al., 2020). But published studies on quality control usually do not cover the entire processing pipeline. In this paper, we outline a processing pipeline for fMRI data that has been used in our lab, and detail the quality control procedure after each of the pre-processing steps. The pre-processing pipeline is suitable for all resting-state, task state, and movie watching conditions (Di and Biswal, 2019, 2020, 2022; Di et al., 2020, 2022a,b). The protocol is based on Statistical Parametric Mapping (SPM) (https://www.fil.ion.ucl.ac.uk/spm/) under MATLAB environment. The quality control issues may be similar when using other major software, e.g., Analysis of Functional NeuroImages (AFNI) (Cox, 1996) and the FMRIB Software Library (FSL) (Jenkinson et al., 2012). But the implementations of quality control in other software are outside the scopes of this paper.

Quality control is mainly 2 fold. The first is to identify artifacts and issues in the images. This includes spatial domain issues, such as ghost artifacts, lesions, and brain coverage, as well as temporal domain issues, such as head motion and other physiological noises. The second is to ensure that the data processing steps can run properly. Practically, many data processing steps rely on iterations, which are sensitive to initial conditions. Quality control is critical to ensure that these processing steps can run properly but are not stuck in local minima. In addition, given the complexity of the fMRI data, there might always be unexpected issues in the images or different processing steps. Visualizations of different aspects of the images will always be helpful to spot the unexpected issues.

Here, we first describe the pre-processing and quality control protocol in detail, including visualizations, exclusion criteria, and the steps needed for processing assurance. The protocol mainly relies on SPM and MATLAB functions. Some visualizations are inspired by previous works, such as TSDiffana (http://imaging.mrc-cbu.cam.ac.uk/imaging/DataDiagnostics) and Power et al. (2014). And secondly, we apply the protocol to the data of the Open QC Project (https://osf.io/qaesm/). We illustrate how quality issues can be identified, and what steps are needed to ensure proper data processing. One particular step is the usage of skull-stripped anatomical images for functional-anatomical images coregistration (Fischmeister et al., 2013). By using the OpenQC dataset, we examine how skull stripping can potentially improve the coregistration compared with using the raw anatomical images.

## 2. Pre-processing and quality control protocol

### 2.1. Software

SPM12: v7771 under MATLAB R2021a environment.

### 2.2. Procedure

**Figure 1:**
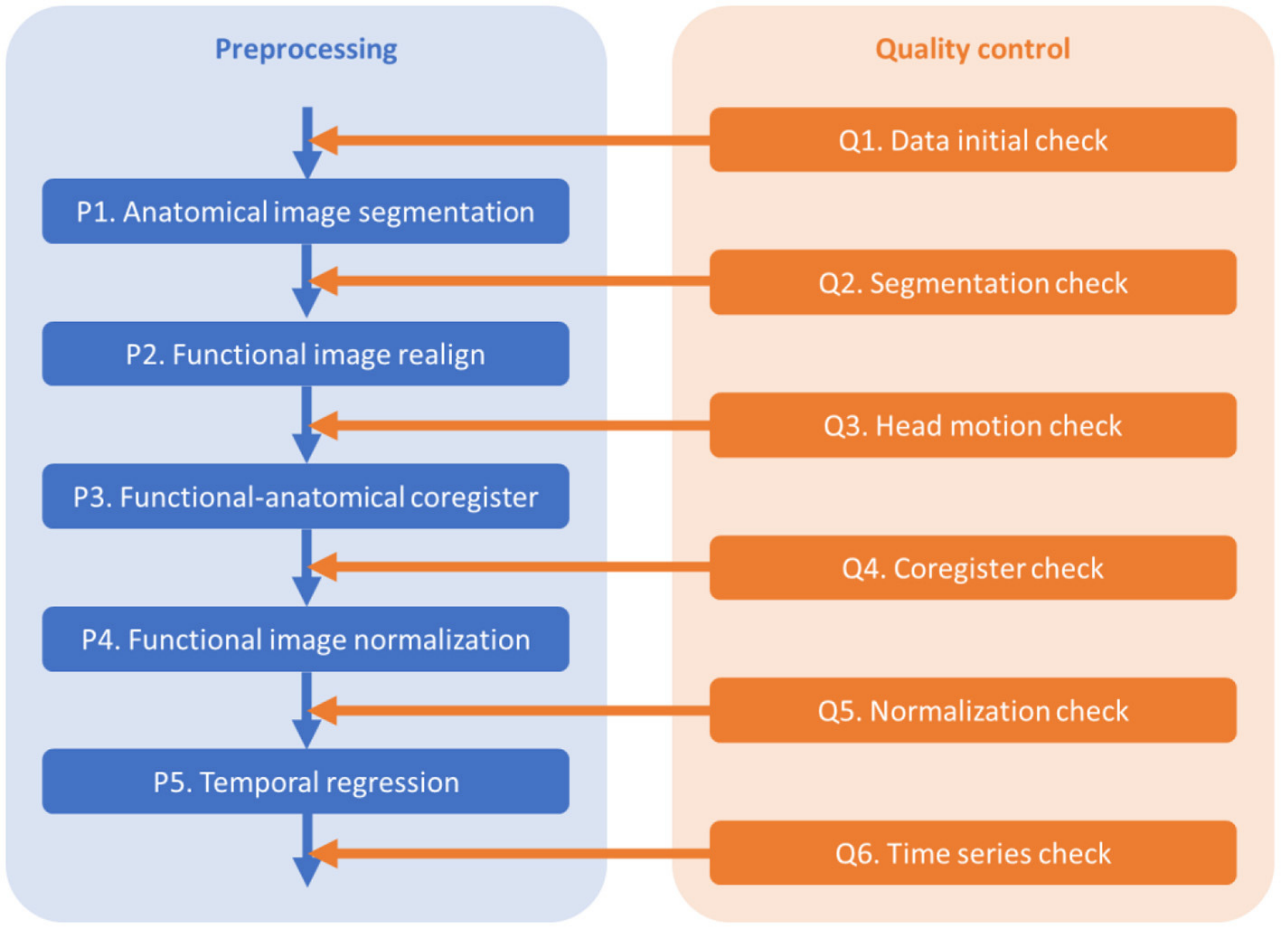
Pre-processing (P) and quality control (Q) steps.

The outline of the pre-processing and quality control steps is shown in Figure 1. The codes are available at https://github.com/Brain-Connectivity-Lab/Preprocessing_and_QC.

#### 2.2.1. Q1. Data initial check

The purposes of the initial check include checking the consistency of imaging parameters across participants, and checking the image quality, coverage, and orientations of the functional and anatomical images.

First, check the key parameters that may affect pre-processing, including the number of volumes, repetition time (TR), and voxel sizes. Plot the parameters across participants (e.g., Supplementary Figure 1) or the histograms may be helpful. If a few participants have different parameters, e.g., fewer volumes, they may be removed from further analyses. If many participants have various numbers of volumes, one may consider keeping the same number of volumes across all the participants. Otherwise, one may also consider including covariates in group level models to account for the parameter variations.

**Figure 2:**
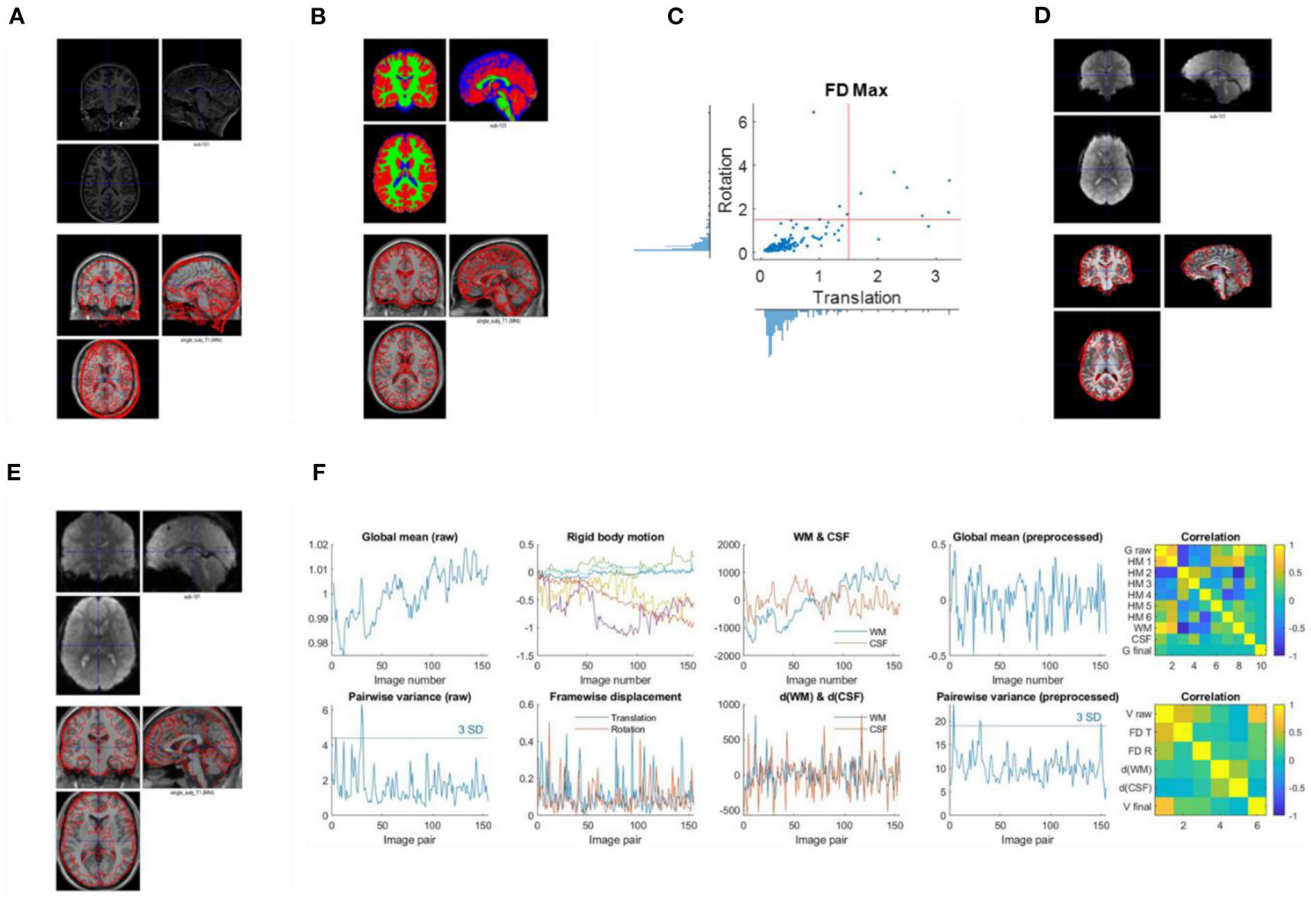
Example visualizations of each quality control step. **(A)** Image initial check (Q1). **(B)** Segmentation check (Q2). **(C)** Head motion check (Q3). **(D)** Coregister check (Q4). **(E)** Normalization check (Q5). **(F)** Time series check (Q6).

Second, check the anatomical images using SPM Check Registration functionality. The first image is the anatomical image of a participant in native space, and the second is the single subject T1 weighted template image in MNI space (Figure 2A). The contour of the first image can be overlayed onto the second image. Focus on, (1) whether the anatomical image has the same orientation and similar initial position to the template, (2) any artifacts, e.g., ghosting, and brain lesion. If any anomaly is noted, then the image needs to be further checked for the whole brain volume. If the anatomical image is located far from the MNI template, or rotated into a different orientation, then manually reorient the image to the template direction and reset the origin to the anterior commissure.

Thirdly, check the first functional image using SPM Check Registration functionality. This is the same as the previous step, except that the first image is a functional image. Focus on (1) whether the functional image has the same orientation and similar initial position to the MNI space template, (2) any artifacts, e.g., ghosting, and (3) the spatial coverage.

#### 2.2.2. P1. Anatomical image segmentation

The purpose of this step is to segment the anatomical image of a participant into gray matter (GM), white matter (WM), cerebrospinal fluid (CSF), and other tissues, and obtain the parameters (deformation fields) for the spatial normalizations of the functional images. A bias corrected anatomical image is also generated, which will be used for functional-anatomical image registration.

Use SPM Segment functionality. The input volume is the subject's anatomical image. Additional non-default setting: (1) “Save Bias Corrected” -> “Save Bias Corrected”; (2) “Warped Tissue” for the first three tissue types (GM, WM, and CSF) -> “Unmodulated”; and 3) “Deformation Fields” -> “Forward.”

DARTEL (a fast diffeomorphic registration algorithm) may be used to generate a sample specific template for spatial normalization (Ashburner, 2007). It can improve cross-individual registrations, especially for a homogeneous sample from a specific population, e.g., children or old adults. But for a large sample size with diverse demographics, DARTEL may not be necessary and is computationally expensive.

#### 2.2.3. Q2. Anatomical image segmentation check

The purpose of this step is to check the quality of segmentation.

Use SPM Check Registration functionality. The first image is the segmented gray matter density image in MNI space (wc1xxx), and the second image is the single subject T1 weighted image in MNI space (Figure 2B). The contour of the first image can be overlayed onto the second image. Next, overlay the segmented images of GM, WM, and CSF (wc1xxx, wc2xxx, and wc3xxx) to the first image.

If misclassification of any tissues is noted, then double check the original anatomical image. If the misclassification could be caused by the position/orientation of the raw anatomical image, try to manually reorient the anatomical image. If brain lesions or image quality issues are noticed, this participant's data should be excluded.

#### 2.2.4. P2. Functional images realign

The purpose of this step is to align all the functional images of a run to the first image. Rigid body head motion parameters (rp files) are also obtained.

Use SPM Realign: Estimate & Reslice functionality. For “Data:Session”: input all the functional images. Non-default setting: “Resliced images”: “Mean Image Only.”

#### 2.2.5. Q3. Head motion check

The purpose of this step is to check the distributions of head motion in the sample, and remove participants with excessive head motions from further analyses.

Calculate framewise displacement (FD) in translation and rotation based on the rigid body transformation results from the P2 step (Di and Biswal, 2015). The formula for FD at time *t* are as follows,


$$\begin{array}{l} FD_{translation,t}\\ =\sqrt{{\left(hp_{x,t}-hp_{x,t-1}\right)}^{2}+{\left(hp_{y,t}-hp_{y,t-1}\right)}^{2}+{\left(hp_{z,t}-hp_{z,t-1}\right)}^{2}}\\ FD_{rotation,t}\\ =\sqrt{{\left(hp_{\alpha ,t}-hp_{\alpha ,t-1}\right)}^{2}+{\left(hp_{\beta ,t}-hp_{\beta ,t-1}\right)}^{2}+{\left(hp_{\gamma ,t}-hp_{\gamma ,t-1}\right)}^{2}} \end{array}$$

Where *hp* represents the head position parameters estimated relative to the first image. *x, y*, and *z* represent the three translation directions, and α, β, and γ represent the three rotation directions. Plot the distributions of maximum framewise displacement across all the participants (Figure 2C). A pre-specified threshold of maximum framewise displacements >1.5 mm or 1.5° (approximately half of the voxel sizes) can be used to exclude participants. However, the threshold may depend on the sample characteristics. See below for more discussions.

#### 2.2.6. P3. Functional-anatomical images coregister

The purpose of this step is to coregister the functional images to the anatomical image of the same individual.

First, generate a skull-stripped bias-corrected anatomical image using SPM Image Calculator (ImCalc) functionality. Input Images: (1) the bias-corrected anatomical image, (2) through (4) c1xxx, c2xxx, and c3xxx segmented tissue images, respectively. Expression: *i*1.^*^ ((*i*2 + *i*3 +*i*4) > 0.5).

Second, use SPM Coregister:Estimate functionality. “Reference Image”: the skull-stripped bias-corrected anatomical image; “Source Image”: the mean functional image generated in the realign step; “Other Images,” all the functional images of the run.

#### 2.2.7. Q4. Coregistration check

The goal of this step is to check the quality of coregistration between the functional and anatomical images.

Use SPM Check Registration functionality. The first image is a functional image of a participant in native space, and second image is the skull stripped anatomical image in native space (Figure 2D). The contour of the first image can be overlayed onto the second image.

Check whether the contour of the functional image aligns with the anatomical image. If the two images are not aligned well, then manual reorientation of the images may be needed.

#### 2.2.8. P4. Spatial normalization

The purpose of this step is to spatially normalize all the functional images into the common MNI space. The normalization parameters are obtained from the segmentation step.

Use SPM Normalize:Write functionality. “Data:Subject:Deformation Field”: y_xxx file from the anatomical image folder; “Images to Write”: all the functional images of a run. Non-default setting, “Voxel sizes”: 3 3 3. The resampling voxel size should be similar to the original voxel size. For the fMRI QC data, we used a common voxel size of 3 × 3 × 3 mm^3^. This may be modified according to the actual voxel size. The resampled voxel size also affects the estimates of spatial smoothness, which may in turn affect voxel-wise statistical results (Mueller et al., 2017).

#### 2.2.9. Q5. Normalization check

The purpose of this step is to check the spatial registrations of the fMRI images to an MNI space template.

Use SPM Check Registration functionality. The first image is the normalized functional image of a participant in MNI space, and the second image is the single subject anatomical image in MNI space. The contour of the first image can be overlayed onto the second image (Figure 2E).

#### 2.2.10. P5. Voxel-wise general linear model

For resting-state data, this step is used to regress out variations of no-interest, such as low-frequency drift, head motion, and WM/CSF signals. The residual images will be further used to calculate functional connectivity or resting-state parameters, such as amplitude of low-frequency fluctuations (ALFF) (Yang et al., 2007), regional homogeneity (ReHo) (Zang et al., 2004), and physiophysiological interaction (PPI) (Di and Biswal, 2013). For task fMRI, the purpose of this step is mainly to derive task related activations.

For resting-state data, firstly, define WM and CSF masks by thresholding and resampling the subject's segmented tissue images using SPM Image Calculator (ImCalc) functionality. “Input Images”: (1) the first functional image (to define the voxel dimension), and (2) wc2xxx or wc3xxx normalized tissue density image. “Expression”: i2 > 0.99. The threshold is used to ensure only WM or CSF voxels are included in the masks.

Secondly, extract the first principal component of the signals in the WM and CSF masks, respectively.

Thirdly, use General Linear Model (GLM) functionality in SPM to perform the regression. The regressors include 24 Friston's head motion model (Friston et al., 1996), the first PC of the WM and CSF, respectively, and a constant term. Note that an implicit high pass filter is also included in the GLM with a cut-off of 1/128 Hz. This GLM step essentially performs artifact removal and filtering together, which can prevent introduced artifacts when doing these two steps separately (Lindquist et al., 2019).

Fourthly, estimate the GLM using SPM Model estimation functionality. Non-default setting, “Write residuals”: Yes.

For task fMRI data, also use the GLM functionality in SPM to perform the regression. Define task regressors using the design timing parameters. Additional regressors include 24 Friston's head motion model (Friston et al., 1996) and a constant term. Note that an implicit high pass filter is also included in the GLM with a cut-off of 1/128 Hz. Next, estimate the GLM using SPM Model estimation functionality. The residual images can be saved to check model fitness, but usually they are not needed for further analyses.

#### 2.2.11. Q6. Time series check

For resting-state data, the purpose of this step is to check the time series of global signals, and their relations to head motion and physiological noises. Mean global signals and pairwise variance [similar to DVARS, temporal derivative of variance (Power et al., 2014)] are commonly used to quality control fMRI time series. Outliers of the variance time series are usually caused by head motion. Therefore, plotting head motion parameters together with the variance and global signals can help to illustrate the relationships. A further question is whether the linear regression step can effectively minimize the noises in the global signals.

Plot time series as Figure 2F. Top row, first, the global mean intensity for the raw fMRI images; second, six rigid body head motion parameters in mm or degree; third, the first PC of the signals in the WM and CSF; and fourth, the global mean intensity for the pre-processed fMRI images within a brain mask. The correlations among all these time series are shown in the last column. Bottom row, first, pairwise variance between consecutive images for the raw fMRI images; second, framewise displacement in translation and rotation; third, derivative (difference) of the first PCs in WM and CSF; and fourth, pairwise variance between consecutive images from the pre-processed fMRI images within a brain mask. The correlations among all these time series are shown in the last column.

The pairwise variance time series is a simple way to spot extreme values. One can use three standard deviations as a criterion to identify the extreme values. Similar spikes can usually be seen in the framewise displacement time series, and sometimes are also visible in the derivatives of the WM/CSF signals. This will result in high correlations among the pairwise variance, framewise displacement, and WM/CSF derivatives. Also focus on the pairwise variance time series from the pre-processed images to check whether they are no longer correlated with the framewise displacement or WM/CSF derivatives. A threshold, e.g., *r* > *0.3*, can be used to identify large correlations.

For task data, the effects of interest are usually the brain activity related to the task design. Then the focus of this step is to check whether the global signals and head motions are correlated with the task design. Therefore, in addition to the time series of global signals and head motion, also plot the task design time series and their derivatives. If the global signals or head motion parameters are correlated with the task design, or the pair wise variance or framewise displacement are correlated with the derivatives of the task design, then one may consider controlling these factors in the first level GLMs.

### 2.3. Other processing steps

Spatial smoothing is not included in this protocol. It is only necessary when voxel-wise statistical analysis is used. If the analysis is ROI based connectivity analysis, then smoothing is not necessary. Moreover, when calculating ReHo, which is a commonly used resting-state measure, the data should also be un-smoothed.

## 3. Materials and methods

### 3.1. Datasets

The data were obtained from the fMRI Open QC Project (https://osf.io/qaesm/). There are anatomical and functional MRI data of 169 participants from eight sites. Seven sites are resting-state fMRI, and the remaining one is task-based fMRI. The data were aggregated from different online resources, including 1,000 Functional Connectomes Project (Biswal et al., 2010), ABIDE (Di Martino et al., 2014), and OpenNeuro (Markiewicz et al., 2021).

The MRI images were acquired using different MRI scanners and imaging protocols. All the MRI scanners were 3T. Table 1 lists some key parameters useful for data analyses. Note that some parameters vary within a site. More imaging parameters for all the participants are shown in Supplementary Figure 1.

**Table 1:** Key imaging parameters in the eight sites of the fMRI Open QC project.

**Site**	***n***	**Number of functional volumes**	**TR (s)**	**Functional image voxel size**			**Anatomical image voxel size**		
				**x**	**y**	**z**	**x**	**y**	**z**
Task	30	242	2	3	3	4	1	1	1
Rest 1	20	128 or 156	2.5	2.67 or 2.29	2.67 or 2.29	3	1	1	1
Rest 2	20	150	2	3	3	3.84	1	1 or 0.93	1 or 0.93
Rest 3	16	162	2.5	1.56	1.56	3.1	0.98	1.2	0.98
Rest 4	23	123	2.5	2.67	2.67	3	1	1	1
Rest 5	20	144	2	3 or 1.85	3 or 1.85	4	1	1	1
Rest 6	20	130–724	2.5	4	4	4	1	1	1
Rest 7	20	198	2.5	3	3	3.51	1	1	1

Shaded cells indicate the presence of different parameters within the site. TR, repetition time.

### 3.2. Pre-processing and quality control

We followed the protocol outlined in Section 2. For each quality control step, an image was saved for each subject. The output images were visually inspected across all the participants. The quality control and exclusion criteria are summarized in Table 2.

**Table 2:** FMRI quality control criteria.

**FMRI quality control criteria**	**Exclude a subject if:**
A. Imaging parameters	Deviating from other participants
B. Anatomical image quality and coverage	Visual assessment
C. Functional image quality and coverage	Visual assessment
D. Segmentation failure	Visual assessment
E. Maximum framewise displacement	>Than 1.5 mm or 1.5°

### 3.3. Data analysis

In the functional-anatomical images coregister step, the current protocol uses the bias-corrected skull-stripped anatomical image as the reference. Because the signals in the skull in the EPI images are weak, in theory it is preferable to coregister the functional images to the skull-stripped anatomical images. However, this is not the default recommendation in SPM. A study has suggested that using the skull-stripped image may improve group-level statistical results (Fischmeister et al., 2013). However, no formal comparison has been performed. We hypothesize that in most cases using the non-skull stripped images will perform the same as the skull stripped images. However, in a small number of cases, using the raw anatomical image may fail. By using the fMRI QC dataset, we estimate the number of cases that would fail when using the raw anatomical image as the reference.

Specifically, we also performed the coregister step by using the raw anatomical image as the reference. We calculated the spatial distance between the functional images to the different reference images. The Euclidean distances were calculated in translation and rotation, separately. We used a threshold of 9 mm or 9° (~3 voxels) to identify cases with excessive differences. We then overlaid the two functional images with the anatomical images to identify potential causes of the discrepancy.

## 4. Results

### 4.1. Q1. Data initial check

Supplementary Table 1 shows some key imaging parameters of the functional and anatomical images for every participant. In resting-state site 1, two participants had fewer fMRI volumes than the rest of the group, which should be removed from analysis. In resting-state site 6, the numbers of fMRI volumes varied between 130 and 724. We kept the first 130 volumes from all the participants for further analysis. The voxel sizes of fMRI images in resting-state site 1 and site 5 varied across participants. Given that only a few participants had different voxel sizes from the majority participants of a site, these participants should be removed from further analysis. The voxel sizes of the anatomical images in resting-state site 2 also varied across the participants. However, it may have minimum impact on the functional images and were therefore were kept for further analysis.

**Figure 3:**
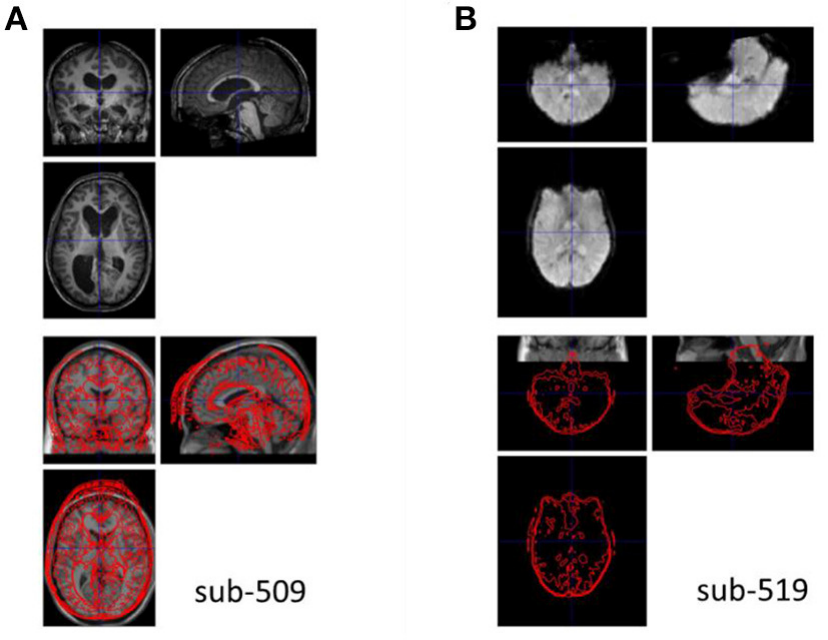
Example anatomical and functional MRI images with quality issues. **(A)** The anatomical image has enlarged ventricle. **(B)** The functional image appears upside down relative to the template image in Montreal Neurological Institute (MNI) space.

The anatomical images were visually inspected for their quality, coverage, and relative positions to the MNI template. All the images were close to the MNI template, indicating that no manual origin setting was needed. One participant's image (sub-509) showed enlarged ventricles (Figure 3A), which should be removed from further analysis. Another participant's image (sub-203, not shown) had mildly enlarged ventricle, which extended to the right lingual territory. We classified this participant as uncertain. This participant may be included if the visual areas were not the main regions of interest.

The quality and coverage of the first fMRI images seemed acceptable for all the participants. However, two participants' images (sub-518, sub-519) appeared upside down (e.g., Figure 3B). The images were manually reoriented to the template orientation.

### 4.2. Q2. Anatomical image segmentation check

**Figure 4:**
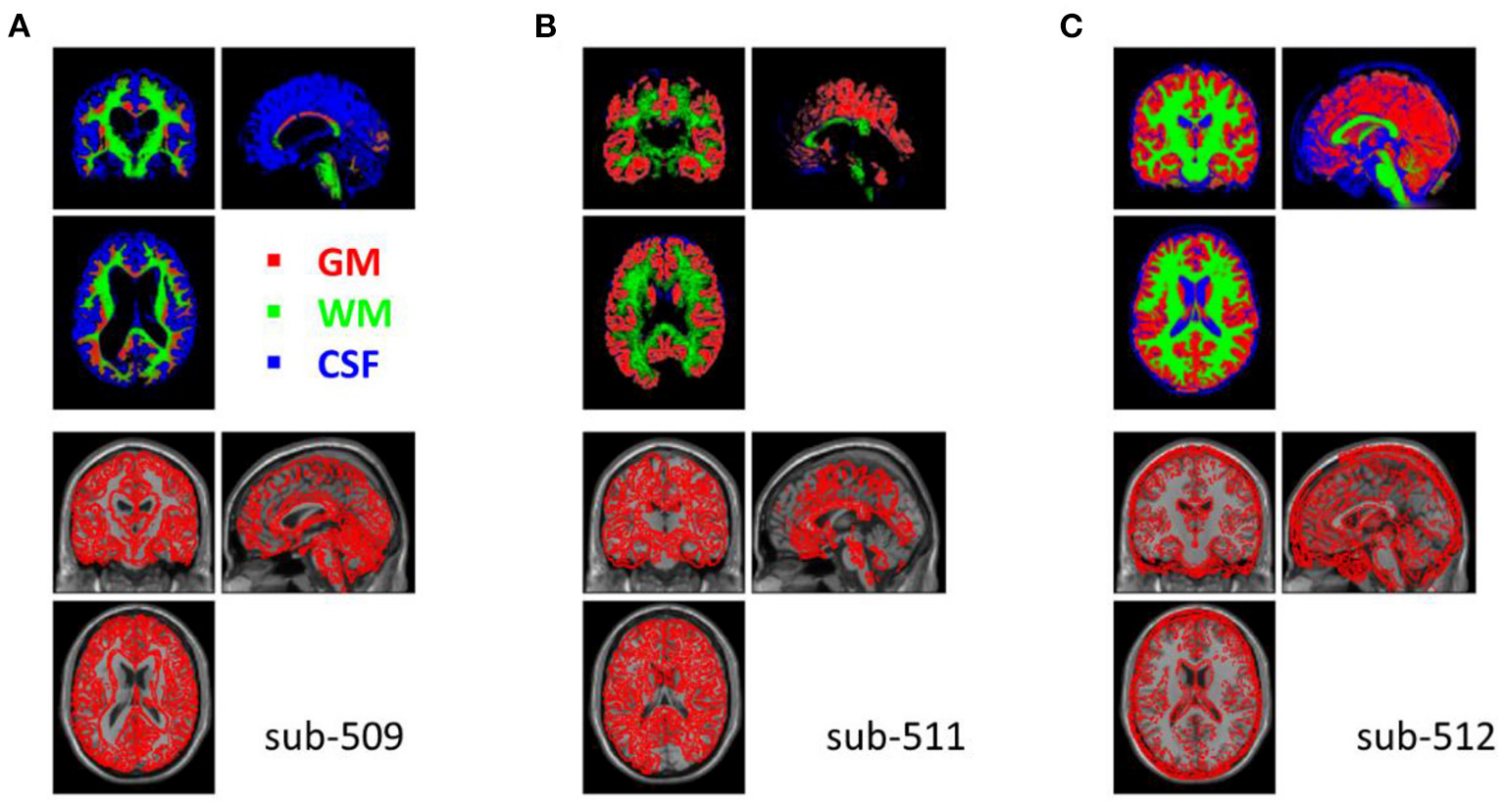
Example anatomical images with segmentation issues. Top row shows segmented tissue images of gray matter (red), white matter (green), and cerebrospinal fluid (blue) in Montreal Neurological Institute (MNI) space. Bottom row shows the single subject T1 image in MNI space with the segmented gray matter contours. **(A)** Shows the participant where most of the gray matter was misclassified as CSF. **(B)** Shows missing classified gray matter in the visual cortex and no classifications of CSF. **(C)** Shows that many soft tissues and bones outside the cortex were miss-classified as CSF.

The segmentation procedure seriously failed in two participants (sub-509 and sub-511). For sub-509, most gray matter regions were identified as CSF (Figure 4A). And for sub-511, part of the visual gray matter was missing, and no CSF was identified (Figure 4B).

Five other participants (sub-108, sub-405, sub-420, sub-512, and sub-514) also have minor segmentation issues, particularly in the CSF (e.g., Figure 4C). Since fMRI analysis usually focuses on gray matter, the misclassifications of CSF may not affect the normalizations of gray matter. These participants may be included in the following analysis. We labeled them uncertain because they may not be included in other types of analysis, such as voxel-based morphometry (Ashburner and Friston, 2000).

### 4.3. Q3. Head motion and variance check

**Figure 5:**
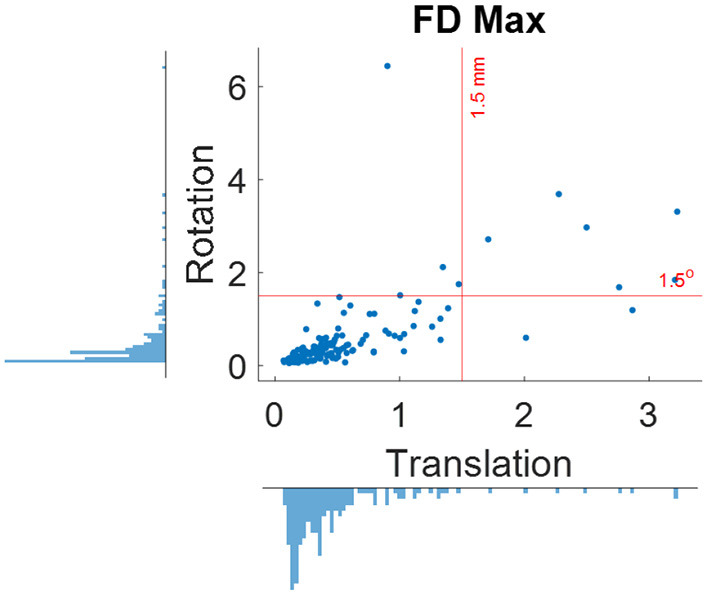
Distributions of maximum framewise displacement (FD) in translation and rotation. The red lines indicate the 1.5 mm and degree thresholds used for excluding participants.

When using the pre-specified threshold of maximum framewise displacement > 1.5 mm or 1.5°, 12 participants were removed from further analysis. Figure 5 shows the distributions of maximum framewise displacement across all the participants. It appears that the 1.5 mm and 1.5° threshold only remove a few participants with excessive head motions. This is desirable because the removal is supposed to only apply to outliers.

### 4.4. Q4. Functional-anatomical images coregister

**Figure 6:**
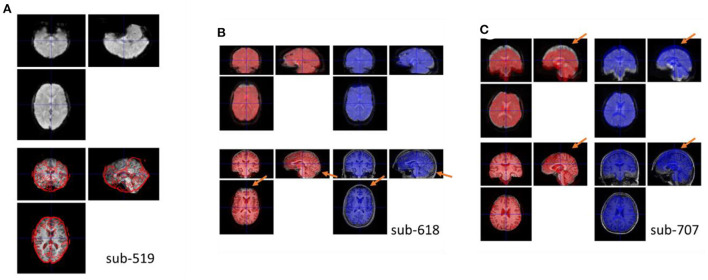
**(A)** Example coregister failure when the functional image was not reoriented correctly. Top row shows the coregistered functional image, and bottom row shows the anatomical image with the contour of the top image. **(B, C)** Example coregister failures when coregistered to the raw anatomical image compared with to the skull-stripped bias-corrected anatomical image. The red brains show the functional images coregistered to the skull-stripped anatomical image, while the blue brain show the functional images coregitered to the raw anatomical image. Top row, the underlay images are the functional images coregistered using the other methods to highlight their differences. Bottom row, the underlay images are the skull-stripped and the raw anatomical images.

For all the participants, the functional images were properly coregistered to their respective anatomical images. This was achieved with the previous quality assurance steps. For example, if the upside-down functional images (sub-518 and sub-519) were not manually reoriented, the coregistration step would fail. Figure 6A shows an example of a functional image registered upside-down with the anatomical image, which was stuck at a local minimum.

Moreover, if the raw anatomical image was used as a reference, the functional images may mis-aligned with the anatomical image in many participants. Figures 6B, C shows two examples. In Figure 6C, the top edge of the fMRI image was aligned to the skull when registered to the raw anatomical image. This is a typical scenario of misalignment. In Figure 6B, the functional image has a signal dropout in the prefrontal region. The distorted prefrontal edge was aligned with un-distorted prefrontal edge in the anatomical image, which resulted in a misalignment. This can be prevented by using the skull-stripped image as the reference. For each participant, we calculated spatial distance in translation and rotation between the functional images coregistered using the two reference images (Supplementary Figure 2). Four participants (2.4%) had spatial distance larger than 9 mm.

### 4.5. Q6. Normalization

All participants' data were successfully normalized into the MNI space.

### 4.6. Q7. Time series check

**Figure 7:**
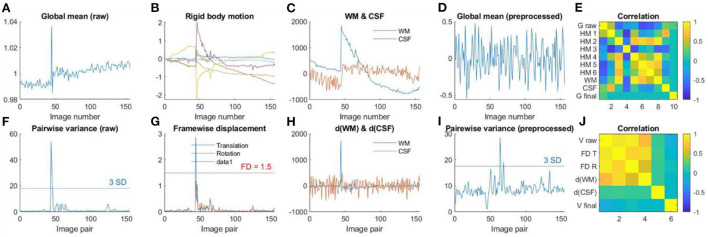
**(A)** Global mean intensity for the raw fMRI images. **(B)** Six rigid-body head motion parameters in mm or degree. **(C)** The first principal component (PC) of the signals in the white matter (WM) and cerebrospinal fluid (CSF). **(D)** Global mean intensity for the pre-processed fMRI images within a brain mask. **(E)** Correlations among **(A)** through **(D)**. **(F)** Variance between consecutive images from the raw data. **(G)** Framewise displacement (FD) in translation and rotation. **(H)** Derivatives of the first PCs in WM and CSF. **(I)** Variance between consecutive images from the pre-processed fMRI images within a brain mask. **(J)** Correlations among **(F)** through **(I)**.

Figure 7 shows an example participant with large head motions. Both the global mean signals (Figure 7A) and pairwise variance (Figure 7F) showed a spike at around the 50th image. The rigid body motion parameters (Figure 7B) and framewise displacement (Figure 7G) showed similar spikes. However, the shapes of the spikes in the rigid body motion parameters appeared different from the global signals (Figure 7A), indicating that simply regressing out the rigid body parameters cannot fully remove motion related noises. In contrast, framewise displacement (Figure 7G) showed strikingly similar patterns as the pairwise variance (Figure 7F). Similarly, the rigid body movement related changes can be seen in the WM signals (Figure 7C), but only the derivatives (Figure 7H) showed similar spike patterns as the pairwise variance (Figure 7F). Next, we check whether the GLM step has successfully minimized the motion related components in the fMRI signals. The global mean signals of the pre-processed images (Figure 7D) no longer contained the spike, and so did the pairwise variance time series (Figure 7I). This is supported by the fact that the pairwise variance from the pre-processed data was not correlated with framewise displacement, which contrasted with the pairwise variance from the raw data (Figure 7J). This suggests that the GLM process can effectively minimize head motion effects in this participant, even though this participant was excluded with our pre-specified threshold.

**Figure 8:**
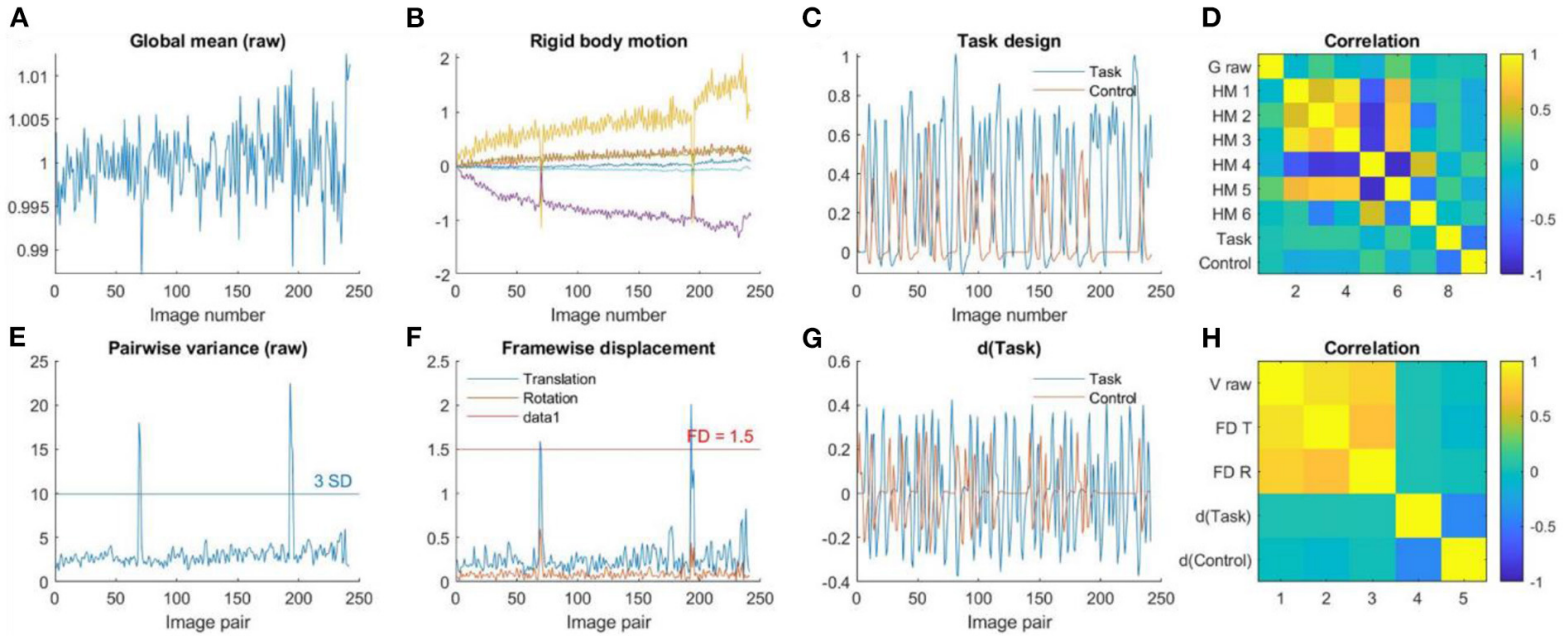
**(A)** Global mean intensity for the raw fMRI images. **(B)** Six rigid-body head motion parameters in mm or degree. **(C)** The task design regressors of the Task and Control conditions. **(D)** Correlations among **(A)** through **(C)**. **(E)** Variance between consecutive images from the raw data. **(F)** Framewise displacement in translation and rotation. **(G)** Derivatives of the task design regressors. **(H)** Correlations among **(E)** through **(G)**.

Figure 8 shows an example participant with large head motions from the task data. The head motion effects were not clearly present in the global mean signals (Figure 8A), but can be clearly seen in the pairwise variance time series (Figure 8E), which can be confirmed in the rigid body motion parameters (Figure 8B) and framewise displacement time series (Figure 8F). For the task-based fMRI, it is critical to verify whether head motion is related to the task design. In Figures 8C, G, we plotted the time series of task design and their derivatives. It seems that head motions were not correlated with the task design, which can be further confirmed in Figures 8D, H.

### 4.7. Summary of quality control results

In total, two participants were discarded due to missing time points; five were discarded due to different fMRI voxel sizes; one was discarded due to poor anatomical image quality; one was discarded due to segmentation failure; and 11 were discarded due to large head motions. Another 5 participants' data had mild issues in the anatomical images or tissue segmentations, which were marked as uncertain. A list of all the excluded or uncertain participants and their reasons is summarized in Supplementary Table 1.

## 5. Discussion

In this paper, we outlined a protocol for fMRI pre-processing and quality control based on SPM and MATLAB. We applied the protocol to the fMRI Open QC dataset, and identified quality issues after each step of pre-processing. We also demonstrated that quality control can ensure proper processing. And specifically, using the skull-stripped anatomical image can help to effectively prevent mis-registrations between functional and anatomical images.

Using a skull-stripped anatomical image as a reference in the coregister step is not the default setting in SPM, but the SPM manual does recommend that if the step is unsuccessful then the skull-stripped images should be used. The current analysis showed that only a small portion of participants have failed this step. However, because they are rare, they are easily overlooked. And in some cases, e.g., Figure 6B, it is not easy to spot the failure visually unless the two functional images are overlayed directly over each other. On the other hand, making the skull-stripped image only takes one simple step with minimal time and computation efforts. Therefore, we recommended that the skull strip should always be performed.

Head motion is a major factor that affect fMRI signals (Friston et al., 1996) and functional connectivity measures (Power et al., 2012; Van Dijk et al., 2012). Different methods have been developed to detect and minimize head motion related artifacts (Friston et al., 1996; Muschelli et al., 2014; Power et al., 2014, 2019). The Friston's 24 model has been shown to be an effective way to reduce motion related artifacts (Yan et al., 2013), which is adopted in the current protocol. In addition to correcting motion related artifacts from the fMRI data, identifying and excluding participants with excessive head motion are also critical. In the current protocol, we set a threshold of 1.5 mm and 1.5° to remove participants with excessive head motions. We note that the threshold is arbitrary. More critically, the distributions of head motion in a sample should always be checked. If the overall head motions are large in the sample, then a more lenient threshold may be considered. If there are multiple groups, e.g., case and control, the distributions of head motion should be compared between groups. Any group differences may need to be controlled in the group-level statistical models. But one needs to keep in mind that excluding participants with large head motion may introduce sampling bias (Kong et al., 2014; Nebel et al., 2022).

Lastly, we note that the quality and formats of fMRI data varied greatly from different sources. We have only demonstrated a handful of quality issues that are present in the fMRI QC project. There are always unexpected issues when processing new data, especially when data are derived from online repositories. Making visualizations of different aspects of the data (e.g., images and time series) is always helpful to ensure proper data processing and to spot unexpected issues.

## Data availability statement

Publicly available datasets were analyzed in this study. This data can be found here: https://osf.io/qaesm/.

## Author contributions

XD performed the analysis and wrote the first draft of the manuscript. XD and BB contributed to conception of the study and manuscript revision, read, and approved the submitted version.
