# Supplementary material for: A functional MRI pre-processing and quality control protocol based on statistical parametric mapping (SPM) and MATLAB
Source: Front Neuroimaging. 2023 Jan 10;1:1070151. doi: 10.3389/fnimg.2022.1070151 (PMC10406300; doi:10.3389/fnimg.2022.1070151)
Supplement: Supplementary file 1 [file Data_Sheet_1.docx]

**Supplementary materials to “A functional MRI pre-processing and quality control protocol based on statistical parametric mapping (SPM) and MATLAB”**

This file contains:

Supplementary Figures S1 and S2

Supplementary Table S1

**Figure S1** Key imaging parameters for all the participants and sites. The vertical black lines separate different sites. For the voxel sizes, the three colors represent x, y, and z directions, respectively. TR, repetition time; TE, echo time.


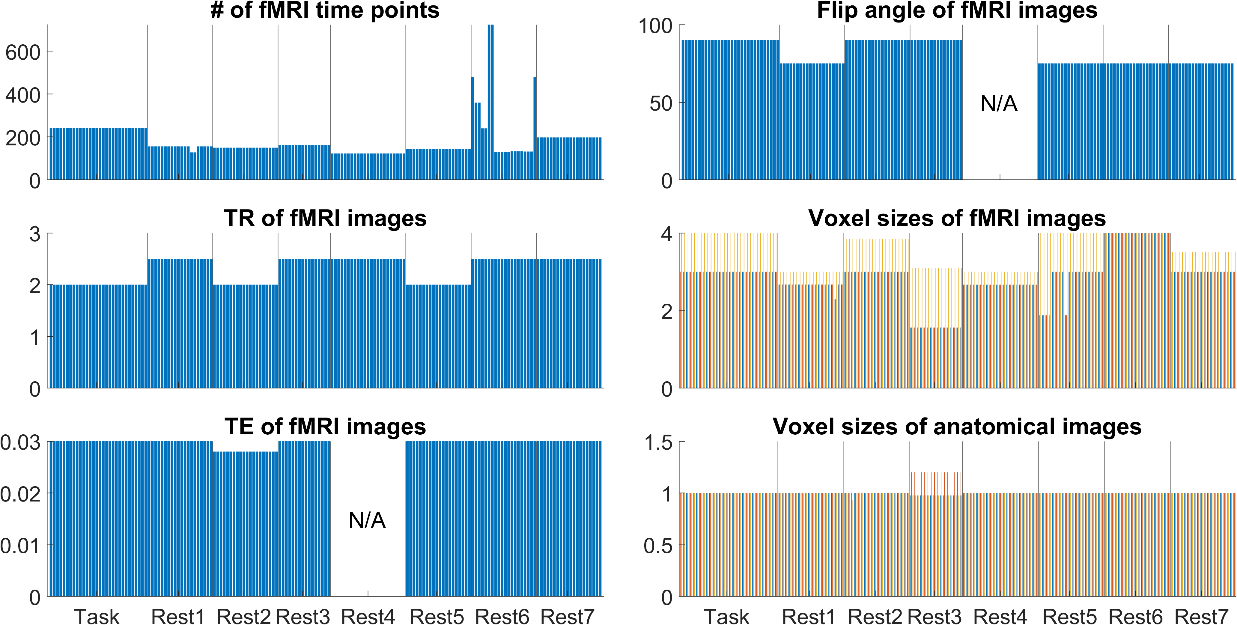


**Figure S2** Spatial distances between the fMRI images coregistered to the raw and skull-stripped bias-corrected anatomical images. The vertical black lines separate different sites.


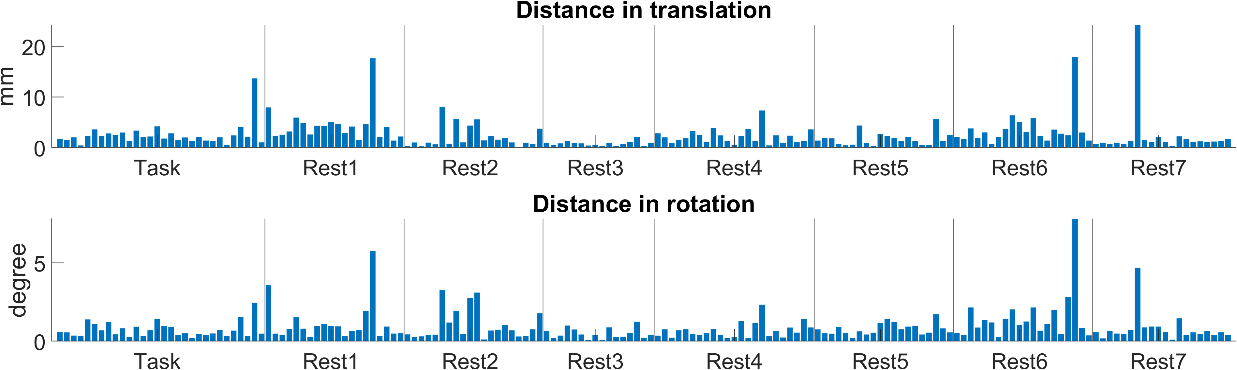


**Table S1** List of all participants who were discarded or uncertain in quality control.

| Participant ID | Exclude | Uncertain | Comment |
| --- | --- | --- | --- |
| sub-012 | x |  | E. Head motion > 1.5 mm or 1.5^o^ |
| sub-017 | x |  | E. Head motion > 1.5 mm or 1.5^o^ |
| sub-022 | x |  | E. Head motion > 1.5 mm or 1.5^o^ |
| sub-029 | x |  | E. Head motion > 1.5 mm or 1.5^o^ |
| sub-102 | x |  | E. Head motion > 1.5 mm or 1.5^o^ |
| sub-107 | x |  | E. Head motion > 1.5 mm or 1.5^o^ |
| sub-108 |  | x | D. Segmentation misclassification in CSF |
| sub-114 | x |  | A. Missing time points |
| sub-115 | x |  | A. Missing time points |
| sub-118 | x |  | A. Different fMRI voxel size |
| sub-119 | x |  | E. Head motion > 1.5 mm or 1.5^o^ |
| sub-203 |  | x | B. Large ventricle |
| sub-207 | x |  | E. Head motion > 1.5 mm or 1.5^o^ |
| sub-405 |  | x | D. Segmentation misclassification in CSF |
| sub-420 |  | x | D. Segmentation misclassification in CSF |
| sub-501 | x |  | A. Different fMRI voxel size |
| sub-502 | x |  | A. Different fMRI voxel size |
| sub-503 | x |  | A. Different fMRI voxel size |
| sub-504 | x |  | A. Different fMRI voxel size |
| sub-509 | x |  | B. Large ventricle |
| sub-510 | x |  | E. Head motion > 1.5 mm or 1.5^o^ |
| sub-511 | x |  | D. Segmentation error |
| sub-512 |  | x | D. Segmentation misclassification in CSF |
| sub-514 | x |  | E. Head motion > 1.5 mm or 1.5^o^ |
| sub-519 | x |  | E. Head motion > 1.5 mm or 1.5^o^ |
